# Percutaneous endoscopic gastropexy for postcolostomy internal hernia of the stomach: a rare case report

**DOI:** 10.1093/jscr/rjag241

**Published:** 2026-04-09

**Authors:** Masahiro Takahashi, Satoru Takayama, Ken Ishikawa, Keisuke Tomoda, Youhei Maeda

**Affiliations:** Department of Surgery, Nagoya Tokushukai General Hospital, 2-52 Kozojicho-kita, Kasugai, Aichi 487-0016, Japan; Department of Surgery, Nagoya Tokushukai General Hospital, 2-52 Kozojicho-kita, Kasugai, Aichi 487-0016, Japan; Department of Surgery, Nagoya Tokushukai General Hospital, 2-52 Kozojicho-kita, Kasugai, Aichi 487-0016, Japan; Department of Surgery, Nagoya Tokushukai General Hospital, 2-52 Kozojicho-kita, Kasugai, Aichi 487-0016, Japan; Department of Gastroenterology, Nagoya Tokushukai General Hospital, 2-52 Kozojicho-kita, Kasugai, Aichi 487-0016, Japan

**Keywords:** internal hernia, colostomy, stomach, percutaneous endoscopic gastropexy

## Abstract

Colostomy-associated internal hernia is a rare postoperative complication, and gastric incarceration through the lateral defect between a lifted sigmoid colostomy and the abdominal wall is exceedingly uncommon, with only three previously reported cases, all of whom were surgically managed for preventing recurrence. We report a rare case of gastric incarceration following sigmoid colostomy in which percutaneous endoscopic gastropexy was performed as a minimally invasive strategy for recurrence prevention. A 59-year-old male presented with vomiting 11 months after undergoing Hartmann’s procedure with an intraperitoneal sigmoid colostomy for sigmoid volvulus. Computed tomography revealed gastric herniation through the lateral defect, causing significant gastric distension. Symptoms resolved following nasogastric decompression, and spontaneous hernia reduction was confirmed. Percutaneous endoscopic gastropexy was subsequently performed without complications. The patient had an uneventful recovery and remained recurrence free at the 4-month follow-up.

## Introduction

Internal hernia represents a rare complication following gastrointestinal surgery, and postcolostomy internal hernia occurring through the lateral defect between the lifted sigmoid colon and the abdominal wall is extremely uncommon. Gastric herniation is particularly unusual, with only three previously reported cases [1–3]. In the reported cases, laparoscopic fixation of the sigmoid colon to the abdominal wall, conversion of the stoma route to an extraperitoneal approach, or laparoscopic gastropexy prevented recurrence. We here report a remarkably rare case of postcolostomy internal hernia of the stomach successfully managed with minimally invasive percutaneous endoscopic gastropexy, effectively preventing recurrence without necessitating additional surgery.

## Case presentation

A 59-year-old male (162 cm, 39.6 kg) presented to our emergency department owing to a 1-day history of persistent vomiting. Eleven months earlier, he had undergone open Hartmann’s procedure with an intraperitoneal lifted sigmoid colostomy for sigmoid volvulus. His past medical history included central cord syndrome, symptomatic epilepsy, hyponatremia, and chronic constipation. He lived in a long-term care facility, ambulated with a walker, and was independent in oral intake. His medications comprised carbamazepine, levetiracetam, zonisamide, olanzapine, lubiprostone, magnesium oxide, famotidine, sodium chloride, limaprost alfadex, and amezinium methylsulfate.

**Figure 1:**
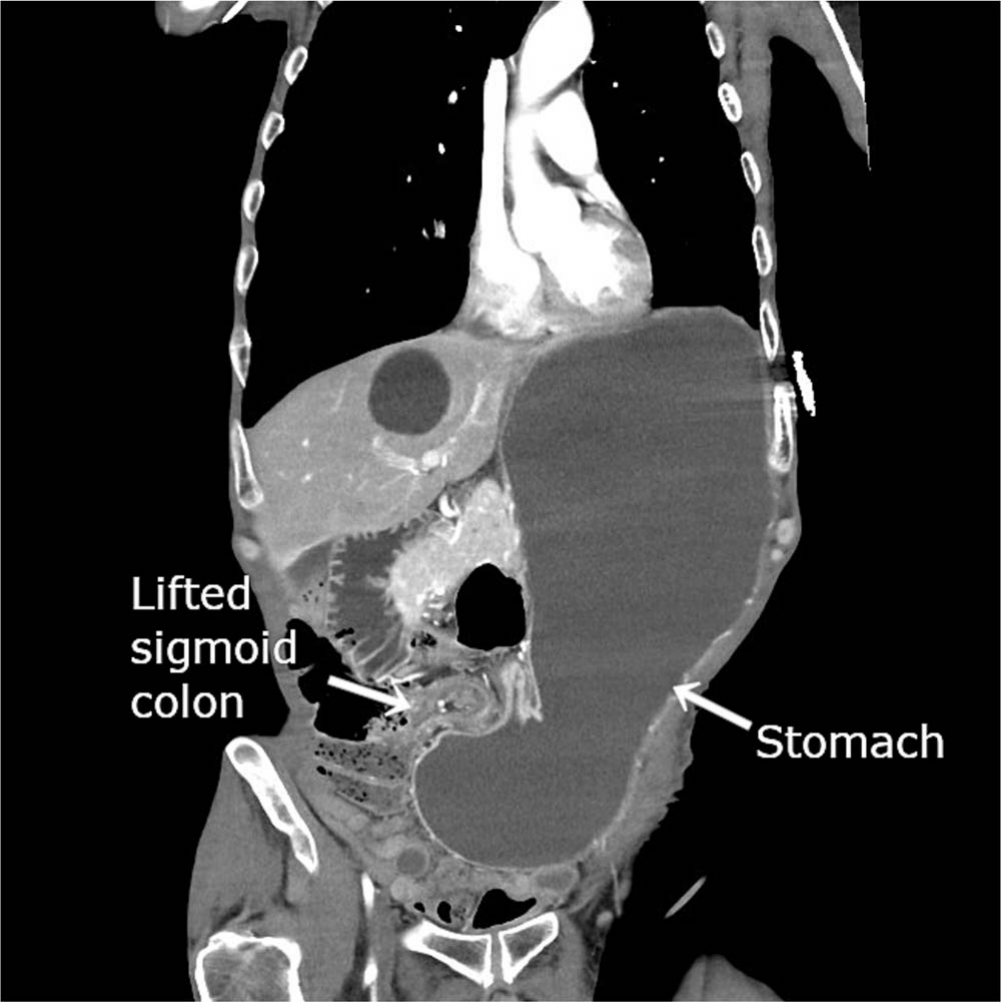
Contrast-enhanced CT revealing gastric herniation through the lateral defect between the lifted sigmoid colon and abdominal wall. The elevated sigmoid colon created for colostomy compresses the pyloric region from the patient’s right side, causing focal narrowing near the antrum. Marked gastric dilatation with significant fluid retention is evident.

He was hemodynamically stable on admission. Laboratory test results revealed leukocytosis (white blood cells, 12 600/μL), elevated C-reactive protein level (2.99 mg/dL), and elevated blood urea nitrogen level (29.7 mg/dL). Contrast-enhanced computed tomography (CT) revealed gastric herniation through the lateral defect between the lifted sigmoid colon and the abdominal wall, causing narrowing near the antrum and marked gastric distension (Fig. 1). Gastric wall enhancement was preserved. Bilateral lung ground-glass opacities and consolidation suggested aspiration pneumonia.

**Figure 2:**
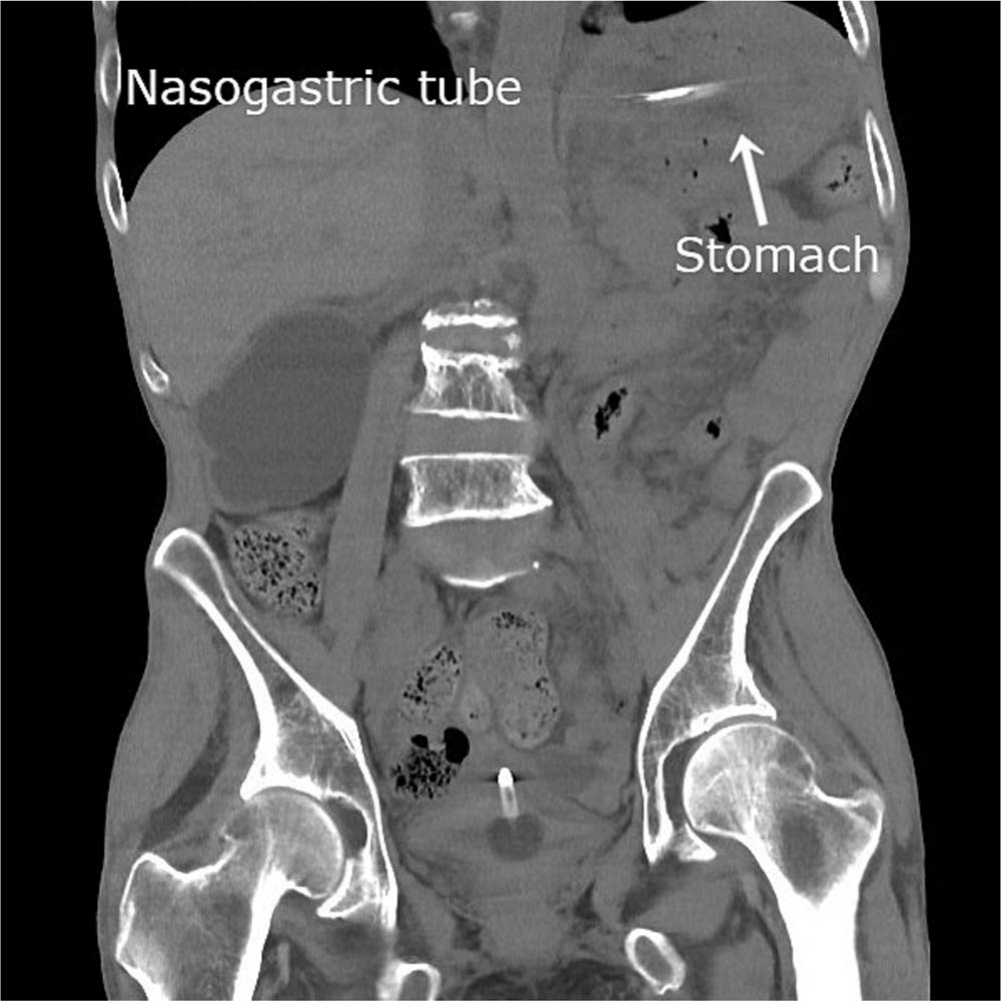
Follow-up plain CT obtained on the day after admission revealing gastric dilatation resolution following nasogastric decompression. The stomach has returned to its normal anatomical position, indicating spontaneous hernia reduction.

The patient was admitted with an initial diagnosis of gastric incarceration due to internal hernia associated with colostomy (IHAC), complicated by aspiration pneumonia. A nasogastric tube was inserted for decompression using intermittent −30 cm H₂O suction, and intravenous sulbactam/ampicillin therapy was initiated. On the same day following gastric decompression, abdominal symptoms resolved. On the day following admission, follow-up CT confirmed gastric distension resolution and spontaneous hernia reduction (Fig. 2). On hospital Day 6, intravenous antibiotics were discontinued following clinical improvement.

**Figure 3:**
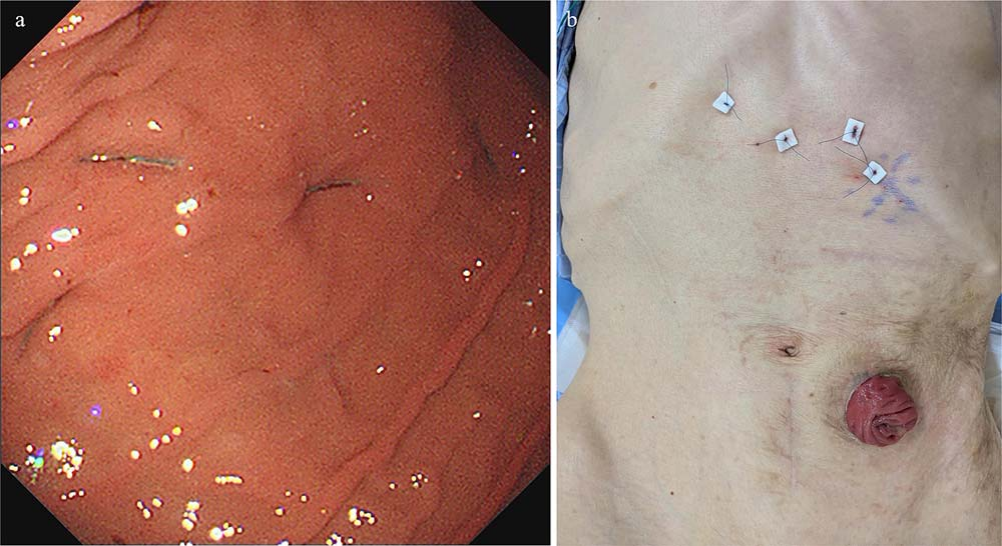
(a) Upper gastrointestinal endoscopic view following percutaneous endoscopic gastropexy revealing four-point fixation at the anterior wall of the mid-gastric body (greater curvature and anterior surface) and at the angular incisure (greater curvature and anterior surface). (b) Postprocedure external abdominal image demonstrating the corresponding four fixation sites.

**Figure 4:**
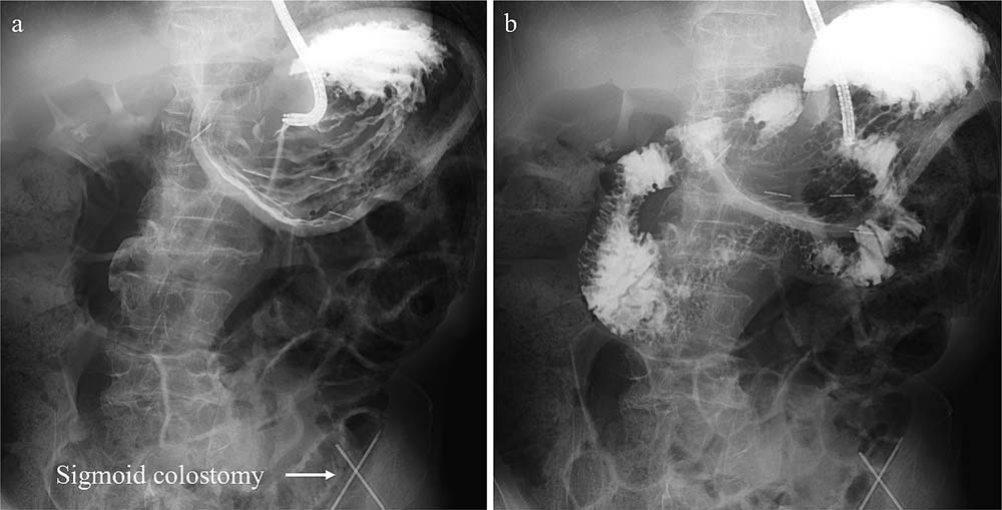
Gastrografin contrast study performed immediately following percutaneous endoscopic gastropexy, (a) fluoroscopic image displaying the normal anatomical location of the stomach with adequate separation from the sigmoid colostomy. (b) Fluoroscopic image showing smooth passage of the contrast medium through the duodenum into the small intestine.

Treatment options for preventing recurrence included laparoscopic repair and percutaneous endoscopic gastric wall fixation, which were discussed with the patient and his family. Minimally invasive endoscopic suture fixation under local anesthesia was selected considering his frailty and comorbidities. The procedure was performed on hospital Day 8 using mild sedation with midazolam (2 mg) and propofol (50 mg). A GIF-XP290N endoscope (Olympus, Tokyo) was introduced with CO₂ insufflation, and upper gastrointestinal endoscopy revealed no mucosal abnormalities. The anatomical association between the stomach and the sigmoid colostomy site was evaluated under fluoroscopic guidance. Through transillumination and finger indentation, the absence of intervening organs between the stomach and abdominal wall was confirmed. Following skin disinfection and local anesthesia with 1% lidocaine, four fixation points were created using a gastrostomy fixation device (Smart Anchor®; TOP Corporation, Tokyo, Japan) with 3–0 nylon sutures at the anterior wall of the mid-body greater curvature, the anterior wall of the mid-body, the greater curvature of the angular incisure, and the anterior wall near the angular incisure (Fig. 3). Adequate separation between the stomach and the sigmoid colostomy and smooth duodenal passage were confirmed on Gastrografin contrast (Fig. 4); the procedure was completed without complications.

Oral intake resumed the following day. On hospital Day 18, he was discharged back to his care facility. At 4-month follow-up, upper gastrointestinal contrast study confirmed stable gastric positioning without recurrence, and he remained asymptomatic.

## Discussion

Stoma stenosis, retraction, prolapse, parastomal hernia, and intestinal obstruction represent major complications following colostomy [4]. Colostomies can be created either through an intraperitoneal or an extraperitoneal route. The intraperitoneal route is frequently selected because it is technically simpler; however, this approach creates a lateral space between the lifted colon and abdominal wall, predisposing patients to IHAC. IHAC is an infrequent complication, and although several cases of small-bowel incarceration or strangulation have been documented [5–7], gastric incarceration is exceedingly rare. To our knowledge, only three cases of IHAC with gastric involvement have been previously reported [1–3]. In all the reported cases, surgical intervention was performed for recurrence prevention. The first, second, and third case was laparoscopically managed by fixation of the sigmoid colon to the abdominal wall, converting the colostomy route from intraperitoneal to extraperitoneal, and laparoscopic gastropexy, respectively.

In the present case, we performed percutaneous endoscopic gastropexy, a less invasive approach that does not require general anesthesia. Conventionally, gastropexy is indicated for gastric volvulus, and currently, it is frequently laparoscopically performed; however, endoscopic techniques have been associated with favorable outcomes [8–15]. Therefore, this approach was considered appropriate for our patient, who had gastric incarceration without bowel ischemia. Techniques for endoscopic gastropexy, including the choice of suture material (absorbable vs. nonabsorbable), number of fixation points, and optimal timing for suture removal, have not been standardized. Previous reports have described the use of both absorbable and nonabsorbable sutures, with nonabsorbable suture removal typically performed between 13 and 42 days postplacement. Early suture removal can diminish local discomfort but may compromise fixation when adhesion between the stomach and the abdominal wall is not yet established. In our case, sutures were removed on postoperative Day 21, a timing that balances comfort and tissue stability.

Unlike gastric volvulus, the direction of torsion does not require consideration when determining fixation points in gastric IHAC. Nonetheless, we used four fixation points for ensuring stability, creating a broad area of adherence. The patient has remained recurrence free at the 4-month follow-up. Alternative surgical strategies, including sigmoid colon fixation or conversion of the colostomy route to an extraperitoneal approach, may be required should recurrence occur.

To our knowledge, this is the first reported case of gastric IHAC successfully managed with percutaneous endoscopic gastropexy. This method may serve as a minimally invasive alternative for preventing recurrence in selected patients. However, as this report describes a single case, conclusions remain limited; further case accumulation is warranted to define optimal management strategies.
